# Evaluation of West Nile Virus Education Campaign

**DOI:** 10.3201/eid1111.050363

**Published:** 2005-11

**Authors:** Ellen Averett, John S. Neuberger, Gail Hansen, Michael H. Fox

**Affiliations:** * University of Kansas School of Medicine, Kansas City, Kansas, USA; † Kansas Department of Health and Environment, Topeka, Kansas, USA

**Keywords:** West Nile virus, West Nile fever, disease prevention, health education, personal protective behaviors, program evaluation, health campaigns, dispatch

## Abstract

We evaluated the 2003 Kansas West Nile virus public education campaign. Awareness was widespread but compliance was low. Spanish-speaking persons were poorly informed. Relevant factors included population segment variability, campaign content, media choice, and materials delivery methods.

West Nile virus (WNV) is a public health problem throughout the United States (*1*). While recovery without sequelae often occurs, the illness can be debilitating and fatal; substantial cost is associated with the illness (*2*). Because no cure or vaccine exists, prevention through education and behavioral change is essential (*3*). Many states have undertaken such prevention efforts, yet their efficacy is unknown, despite the Centers for Disease Control and Prevention's (CDC) request for evaluations (*4*). Although some states have assessed residents' knowledge, attitudes, and behaviors about WNV (*5**–**9*), Kansas is the first to report a process and outcome evaluation of its education campaign.

In 2003, the Kansas Department of Health and Environment (KDHE) implemented an extensive WNV prevention education campaign. Based on CDC recommendations, the campaign focused on education and encouraged 4 preventive behavior measures: use insect repellent with DEET (N, N-diethyl-meta-toluamide), wear long-sleeved shirts and long pants when outside at dawn or dusk, eliminate standing water, and maintain window screens.

Campaign materials were developed for television, radio, newspapers, and a web site. Public service announcements (PSAs) for print and broadcast media and brochures in English and Spanish were produced. Materials were distributed by email and the US Postal Service well before expected summer WNV outbreaks.

## The Study

Evaluation focused on a sample of 10 representative counties and consisted of personal and media surveys. The University of Kansas Medical Center Human Subjects Committee approved the surveys. The personal survey assessed knowledge, behavior, and attitudes about WNV (*8*). Knowledge was measured by asking respondents how WNV is transmitted, who is most vulnerable, and what self-protection measures are available. Behavior was measured by asking what respondents had done during the past week to protect themselves from mosquito bites. Attitude was measured by asking respondents their perceived risk of contracting WNV and for concerns or comments about recommended protective measures.

Surveys were conducted from August through October. Respondents were chosen from randomly generated telephone numbers. Telephone calls were made from 9:00 a.m. to 8:00 p.m. Monday through Saturday. The survey was administered in Spanish if respondents preferred to speak Spanish. A total of 2,329 calls were made reaching 779 eligible respondents; 534 (69%) respondents participated in the survey. Compared to Kansas' Census 2000 data, the sample's demographics were comparable to the general population.

Ninety seven percent of the sample had heard of WNV, 94% knew that it is transmitted through mosquitoes, and 70% knew that persons >50 years of age were most likely to become severely ill from it. Among the 17 Spanish-speaking respondents, there was significantly less awareness (p<0.001, chi-square test); only 7, (41%) had heard of WNV.

Among respondents who had heard of WNV, 89% knew >1 personal protective measure, 59% knew to avoid mosquitoes, 47% knew to use insect repellent, and 21% specified the use of repellent with DEET (Table 1). Reported behavior (Table 1) did not reflect knowledge. Fewer respondents used repellent than those who had cited this measure (p<0.001, chi-square test). More respondents wore protective clothing (p<0.001, chi-square test), eliminated standing water (p<0.001, chi-square test), and maintained window screens (p<0.001, chi-square test) than those who had cited these measures.

**Table 1:** Comparison of respondents' knowledge and behavior about West Nile virus (WNV) protective measures*

WNV personal protective measures	% of respondents who knew specific WNV personal protective measures	% of respondents who took specific WNV or mosquito-bite protective measures in past week
Use repellent	47.2	27.5†
Use DEET repellent	21.3	17.6
Wear long clothes	26.6	36.9†
Eliminate standing water	34.3	54.1†
Repair window screens	.6	33.0†
Other measures‡	59.2	NA

*DEET, N, N-diethyl-meta-toluamide; NA, not available. †p<0.001, chi-square test. ‡Mostly general mosquito avoidance.

To understand protective measures attitudes, respondents were asked for comments or concerns. More than one third expressed concerns about DEET, mostly about health and safety; 26% stated that wearing long clothing outside at dawn and dusk was uncomfortable, and 16% cited difficulties eliminating standing water. Respondents estimated the risk of contracting WNV; 55% considered the risk to be low and 8% considered it to be high. These data are consistent with other studies (*5**–**9*).

To assess the process and outcome of the KDHE campaign, respondents were asked to list their most recent sources of WNV information. Most respondents cited mass media and word-of-mouth, few cited magazines or web sites, and even fewer cited healthcare providers or brochures (Table 2).

**Table 2:** Percentage of respondents citing various media as sources of West Nile virus information

Source	%
Television	88
Newspaper	72
Word-of-mouth	65
Radio	44
Magazine	17
Website	16
Veterinarian	9
Healthcare provider	8
Brochure	6
Other	5

Process and outcome were also evaluated through the media survey. Every newspaper, radio station, and television station in the 10-county sample was contacted by phone or email. All were asked about receipt of KDHE WNV materials and if and how materials were used. Results indicated minimal use of the materials. No television station broadcasted PSAs or scripted stories provided by KDHE; only half recalled receiving the materials. Fifteen of 40 radio stations recalled receiving materials, although none aired them. Ten of 23 newspapers recalled receiving materials; 5 used the materials in publication.

## Conclusions

Most persons were knowledgeable about WNV and cited mass media as their information source. KDHE campaign materials were used minimally by local media; persons were likely informed from national news, CDC, or news releases when local cases of WNV were reported. Low awareness levels in Spanish-speaking respondents indicated that prevention messages from any source were not reaching this population segment.

Three factors appeared to influence the degree to which WNV prevention messages affected respondents' knowledge, attitudes, and behavior: message content, media used, and method of delivery. More respondents knew they should be using repellent than actually used it. Knowledge and awareness are insufficient to impact behavior. Risk perceptions may be a moderating factor. Despite 90 confirmed cases of WNV, a 4-fold increase from 2002, and 731 reported presumed cases, most respondents perceived little risk of acquiring the illness. Thus, they likely were not motivated to use protective measures, especially those seen as deleterious or unpleasant. More respondents took other protective measures (wore protective clothing, removed standing water, maintained screens) than cited them. Clearly some took these measures for other reasons, unaware that they provided protection from WNV.

Mass media and word-of-mouth were the most successful methods of providing WNV information to respondents. Healthcare providers, veterinarians, magazines, and the Internet were less successful methods. Brochures were least successful, indicating that they may be ineffective for this type of communication, or that difficulties with their distribution occurred.

Timing and campaign material delivery methods were critical factors. Email delivery was problematic; some attached files could not be opened. Some broadcast stations had policies prohibiting opening unsolicited email attachments. Timing of materials distribution also contributed to their minimal use. Materials were sent in the spring before the peak of WNV incidence. By the time our survey was conducted during peak WNV season, news media considered WNV a "hot topic." Media respondents often did not recall receiving WNV materials, yet asked our surveyors for materials. News media look for current materials and do not likely store information for later use.

We recommend the following practices for public health disease prevention campaigns for WNV and other emerging diseases: 1) Reduce barriers to desired behavioral changes. For WNV, this includes greater focus on the safety of DEET. 2) Distribute materials by email with provisions to assure recipients that materials are virus-free. Send explanatory letters by the postal service before and after any email. Provide a a web site for direct access to campaign materials. 3) Provide information close to the time it will be used. 4) Research ways to influence and promote word-of-mouth, an important source of information. 5) Research how media differentially impact population segments. 6) Increase PSA exposure; consider purchasing broadcast time. 7) Design campaigns for a linguistically and culturally diverse population (*10*) and to reach vulnerable population subgroups such as the elderly and immunocompromised for WNV (*11**–**15*).
